# 

**Published:** 1882-01

**Authors:** 


					﻿JANUARY, 1882.
TWENTY-NINTH YEAR.
1IAI.IJS
JOURNAL
or
HEALTH'
E. H. GIBBS, A.M., M.D., Editor,
No. 141 EIGHTH STREET (Near Broadway), NEW YORK.
1 TERMS: $2 a Year, or $1 for Six Months. Single nnmlier, 20 cts.
				

